# Long-term outcome of Graves’ orbitopathy following treatment with sirolimus

**DOI:** 10.1007/s40618-024-02470-8

**Published:** 2024-10-07

**Authors:** Simone Comi, Giada Cosentino, Giulia Lanzolla, Francesca Menconi, Maria Novella Maglionico, Chiara Posarelli, Francesco Latrofa, Roberto Rocchi, Michele Figus, Ferruccio Santini, Michele Marinò

**Affiliations:** 1 Department of Clinical and Experimental Medicine, Endocrinology Units, University of Pisa, Via Paradisa 2, 56124 Pisa, Italy; 2 Department of Orthopaedic Surgery, University of Pennsylvania, Philadelphia, PA USA; 3 Department of Surgical, Medical and Molecular Pathology, Ophthalmopathy Unit I, University of Pisa and University Hospital of Pisa, Via Paradisa 2, 56124 Pisa, Italy

**Keywords:** Sirolimus, Graves’ disease, Graves’ orbitopathy, Rapamycin, Thyroid eye disease, Thyroid autoimmunity

## Abstract

**Objectives:**

Sirolimus was found to be associated with a better outcome of Graves’ orbitopathy (GO) at 24 weeks compared to methylprednisolone. We conducted a retrospective study to investigate its efficacy and safety over a longer period.

**Methods:**

Data from 40 consecutive patients with moderate-to-severe, active GO, 20 treated with sirolimus and 20 with methylprednisolone, were collected. Primary outcome: overall outcome (composite evaluation) of GO at 48 weeks. Secondary outcomes: (1) GO outcome at 24 weeks, and, at 24 and 48 weeks: (2) outcome of single eye features; (3) quality of life (GO-QoL); (4) TSH-receptor antibodies; (5) GO relapse at 48 weeks; (6) adverse events.

**Results:**

The overall GO outcome at 48 weeks did not differ between the two groups (responders: 55% vs 55%). At 24 weeks, prevalence of responders was greater in sirolimus group (65% vs 25%; P = 0.01). A reduction ≥ 1 point in clinical activity score (CAS) was more frequent in sirolimus patients at 24 (85% vs 40%; P = 0.005) and 48 weeks (75% vs 60%; P = 0.03). The proportion of GO-QoL responders (appearance subscale) at 24 weeks was greater in sirolimus group (62.5% vs 26.3%; P = 0.03). No difference was observed for the remaining outcome measures.

**Conclusions:**

Treatment with sirolimus is followed by a greater overall response of GO compared with methylprednisolone at 24 weeks, but not at 48 weeks, when only CAS is affected. A more prolonged period of treatment may be required for a better outcome to be observed over a longer period.

## Introduction

Graves’ orbitopathy (GO) is the most prevalent extrathyroidal manifestation of Graves’ disease (GD), being observed in ~ 25% of GD patients [1]. The pathogenesis of GO is believed to reflect an autoimmune process [2]. The thyroid stimulating hormone receptor (TSHR), which is expressed by orbital fibroblasts, is believed to be recognized by autoantibodies and/or autoreactive T-cells, and to interact with the insulin-like growth factor receptor (IGF-1R) [3], resulting in activation of both receptors, and, finally, in fibroblast growth, adipogenesis and secretion of glycosaminoglycans [2, 3]. These events are to some extent elicited also by cytokines secreted by immune-competent cells and by fibroblasts themselves, as well as by the oxidative stress in the orbital microenvironment [2].

The 2021 Guidelines of the European Group On Graves’ Orbitopathy (EUGOGO) recommend high-dose intravenous glucocorticoids (ivGCs) as the first-line treatment for moderate-to-severe, active GO [4]. However, the response rate to ivGCs is highly variable, ranging from 25 to 90% [5, 6], depending on dosage, number of pulses, duration of treatment and response measures. Resistance to GCs is a known phenomenon that can be ascribed also to genetic reasons. In this regard, the observation that GO only rarely disappears completely after treatment [7], has been postulated to reflect epigenetic mechanisms [8].

The recent acquisitions on the pathogenetic mechanisms have allowed the identification of novel medications for GO [4]. Among them, teprotumumab, a monoclonal anti-IGF-1 receptor (IGF-1R) antibody, resulted to be very effective [9, 10]. However, its high cost and its toxicity could limit an extensive use [11, 12].

Sirolimus (rapamycin) is an immunosuppressive drug with anti-proliferative and anti-fibrotic properties, used to prevent graft rejection after kidney transplantation in adult patients with a mild to moderate immunological risk; in addition, sirolimus is approved for lymphangioleiomyomatosis and for medicated stents in patients undergoing coronary angioplasty [13–15]. The rationale behind the use of sirolimus lies in its mechanisms of action. By inhibiting the calcium-dependent and calcium-independent translation signals, sirolimus blocks T-cell activation. In addition, by forming a molecular complex with FKBP12 protein, the drug binds to the mammalian target of rapamycin (mTOR) and consequently inhibits mTORC1 (mTOR complex-1) [15]. This results in a reduction of CD4- and CD8-positive T cells activity and trafficking [16, 17], in a decreased production of proinflammatory cytokines, especially IL-16 [18–20], and in an inhibition of both adipogenesis and differentiation of fibroblasts into myofibroblasts [20–24]. Interestingly, by acting downstream of IGF1-R, the effects of sirolimus on orbital fibroblasts partially overlap those of teprotumumab [20].

The most common side effects of sirolimus include urinary tract infections, alterations of the hydro-electrolyte balance, lipid profile impairments, gastrointestinal symptoms, anemia, thrombocytopenia, arthralgia, peripheral edemas and hypertension [13, 14]. However, when used at low dosage, adverse events are uncommon [13, 14]. Furthermore, sirolimus is not nephrotoxic and dosage adjustments are not required in patients with renal failure [13, 14].

Two case reports of patients with GO resistant to glucocorticoids and successfully treated with sirolimus were previously described [25, 26]. Based on the knowledge of its mechanisms of action and on these case reports, we started using sirolimus, given off-label at low dosage as a second-line treatment, in patients with persistence or recurrence of moderate-to-severe, active GO. Results from the first 15 patients treated with sirolimus showed a greater overall response at 24 weeks compared with patients given a second course of methylprednisolone [27]. These results were associated with a fairly good safety profile of the drug [27]. Here we investigated retrospectively, over a longer period of time, the efficacy and safety of sirolimus compared with methylprednisolone in patients with moderate-to-severe, active GO.

## Subjects and methods

### Study design

We conducted a retrospective, single-centre, no-profit, clinical study aimed at assessing the long-term effects of sirolimus, used as second-line treatment, on the outcome of moderate-to-severe, active GO compared to ivGCs. The research design entailed the inclusion of all consecutive patients treated with either sirolimus or methylprednisolone over 34 consecutive months.

### Setting

The study was performed in a Tertiary Referral Center, namely the University Hospital of Pisa. The study was approved by the local Ethic Committee (Comitato Etico Area Vasta Nord-Ovest, approval no. 21672_MARINO) and performed in accordance with the International Conference on Harmonisation Good Clinical Practice guidelines and the principles of the Declaration of Helsinki.

### Participants

Inclusion criteria were: (1) men and women aged between 18 and 75 years; (2) moderate-to-severe, active GO, defined by the presence of a Clinical Activity Score (CAS) ≥ 3 on a 7-point scale associated with at least one of the following criteria in the most affected eye: (i) exophthalmos ≥ 2 mm compared with normal for gender and race; (ii) inconstant or constant diplopia; and (iii) lid retraction ≥ 2 mm; (3) written, signed informed consent, including compliance with requirements and restrictions listed in the consent form; and, only for the sirolimus group: (4) previous treatment with ivGCs, administered more than 24 weeks prior to starting sirolimus.

Exclusion criteria were: (1) optic neuropathy, as defined by the 2021 EUGOGO guidelines [4]; (2) treatment with GCs or other immunosuppressive medications, and/or selenium within the 24 weeks prior to the current treatment; (3) previous orbital radiotherapy; (4) previous orbital decompressive surgery; (5) mental illness preventing comprehensive informed consent.

Signed informed consent was obtained from all patients.

### Outcomes

The primary objective of the study was the overall outcome of GO at 48 weeks, based on a composite evaluation, as recommended by the 2021 EUGOGO guidelines [4, 28]. Overall response was defined as the presence of at least two of the following criteria in the most affected eye, without worsening in any of the same measures in the other eye: (1) reduction ≥ 1 point in 5-scale CAS (spontaneous and gaze-evoked pain excluded); (2) reduction ≥ 2 mm in exophthalmos; (3) reduction ≥ 2 mm in eyelid aperture; (4) increase ≥ 8° in the sum of eye muscle ductions.

Secondary objectives were: (1) overall GO outcome at 24 weeks; at 24 and 48 weeks: (2) outcome of single eye features, with the addition of diplopia (disappearance or improvement, namely change from constant to inconstant, intermittent, or absent, or from inconstant to intermittent or absent); (3) outcome of quality of life (QoL) [increase ≥ 6% of GO-specific QoL questionnaire (GO-QoL) score]; (4) TSH-receptor antibodies (TRAbs); and (5) GO relapse at 48 weeks (prevalence of patients with a worsening of at least two of any eye features from 24 to 48 weeks).

Safety monitoring was performed. Adverse events were documented and graded according to National Cancer Institute Common Terminology Criteria for Adverse Events (CTCAE), version 5.0.

### Procedures

Patients in the sirolimus group were treated with a loading dose of sirolimus of 2 mg orally on the first day, given on fasting, approximately at 10 am, followed by 0.5 mg per day for 12 weeks. Patients in the methylprednisolone group received intravenous methylprednisolone according to the following, previously described [4], protocol: 500 mg weekly for 6 weeks, 250 mg weekly for the subsequent 6 weeks (cumulative dose: 4.5 g). Within the methylprednisolone group, all patients were given omeprazole 20 mg/daily across the treatment period to prevent gastrointestinal adverse events, whereas post-menopausal women were treated with bisphosphonates (i.e. alendronate 70 mg/week) and vitamin D (i.e. cholecalciferol 25.000 IU/month) to prevent bone loss.

### Sources of data and measurements

All patients underwent an ophthalmological assessment at baseline, 24 and 48 weeks, which included the following procedures: (1) exophthalmometry (Hertel exophthalmometer); (2) measurement of eyelid aperture; (3) assessment of diplopia (Gorman score) [4]; (4) ocular ductions; (5) assessment of the corneal status; (6) examination of the fundi; (7) visual acuity (Snellen chart); and (8) evaluation of CAS [29]. Patients were evaluated by two ophthalmologists (M.N.M, C.P) at the same time at all visits, in order to minimize inter- and intra-observer variations.

The following blood tests were performed at baseline and every four weeks up to 24 weeks: free thyroxine (FT4), by chemiluminescence immunoassays (Vitros Immunodiagnostics, Raritan, NJ); thyroid stimulating hormone (TSH), by immunochemiluminometric assay (Immulite 2000, Siemens Healthcare, Gwynedd, UK). TRAbs were measured at baseline, 24 and 48 weeks by enzyme-linked immunoassay (ElisaRSR™ TRAb 3rd Generation, Cardiff, UK). The following blood tests were performed at baseline and every two weeks up to 24 weeks: blood count, sodium, potassium, phosphate, creatinine, aspartate aminotransferase, alanine aminotransferase, creatine phosphokinase, alkaline phosphatase, fasting blood glucose, total cholesterol, low-density lipoprotein-cholesterol, high-density lipoprotein-cholesterol, triglycerides, all by enzymatic-colorimetric assays (Roche, Mannheim, Germany), urinalysis by automated urine chemistry analyzer (UNIMAX, Menarini, Firenze, Italy) and automated urine sediment analyzer (sediMAX conTRUST PRO, Menarini, Firenze, Italy), and urine culture by calibrated loop method (Sidecar, Alifax, Polverara, Italy). Rapamycin was measured in the sirolimus group at 12 weeks, 2–3 days prior to the end of treatment, by chemiluminescent microparticle immunoassay (ARCHITECT Sirolimus, Abbott, Chicago, Illinois, USA).

A specific questionnaire for GO was used to assess QoL [30]. Questionnaire comprises two subscales: (1) visual functioning (eight questions concerning limitations attributable to decreased visual acuity, diplopia, or both), and (2) appearance (eight questions referring to limitations in psychosocial functioning attributable to changes in appearance). Questions are scored as severely limited (one point), a little limited (two points), or not limited at all (three points). The total score as well as the two subscales were converted into percentages according to the following formula: (total points*100)/(number of questions answered*3). A higher percentage means a better QoL. Patients filled the questionnaire at baseline, 24 and 48 weeks. Patients were defined responders when an increase by at least 6% from baseline occurred.

Data were collected and recorded in a database. The following database validation procedures were employed: allowed character checks, batch totals, missing records check, cardinality check, digits check, consistency check, control totals, cross-system consistency check, data type check, hash totals, limit check, logic check, presence check, range check, spelling and grammar check, and uniqueness check.

### Study size

In a previous study, conducted in 15 patients per group, we observed 86.6% of responders in patients given sirolimus *vs* 26.6% in patients given methylprednisolone at 24 weeks [27]. Assuming that similar results would have been observed at 48 weeks, we estimated that a total of 18 patients would have been sufficient for statistical significance with P < 0.05 by Fisher’s exact test for primary outcome in the final analysis, with a statistical power of 0.8. Thus, the number of patients studied, namely 40, largely exceeds that estimate.

### Statistical analyses

Continuous variables are presented as mean (SD) or median (IQR), as appropriate, and differences were assessed by ANOVA with Bonferroni’s correction or Mann–Whitney. Categorical data were compared by two-tailed Fisher’s exact test. The impact of the baseline features of the two groups on the study outcomes was assessed by binary logistic regression. Analyses were performed using SPSS version 21.0 (IBM, New York, NY).

## Results

Between March 15th 2021 and January 15th 2023, a total of 20 patients with moderate-to-severe and active GO were treated with sirolimus. Sirolimus was given as second-line treatment, having all patients previously been treated with a course of methylprednisolone (cumulative dose: 4.5 g), which was followed, in all cases, by persistence or recurrence of GO. Sirolimus was given off-label because of contraindications to ivGCs (7 patients with severe non-alcoholic fatty liver disease, 4 patients with severe osteoporosis), or because patients refused a second course of ivGCs. The control group comprised 20 patients treated with methylprednisolone over the same period. None of the patients of the present study participated in our previous study on sirolimus [27].

Table 1 shows demographic and clinical features of the two groups at baseline. All patients given sirolimus and none in the methylprednisolone group had been previously treated with ivGCs. As a consequence, sirolimus group had a greater age and GO duration compared to methylprednisolone group. As expected from patients with a longer GO duration, patients given sirolimus had more frequently received definitive treatment for hyperthyroidism. Finally, concerning GO-QoL, patients given sirolimus had a significantly lower total score and a trend to a lower functioning subscale score, as expected in patients in whom a prior course of ivGCs had failed. No difference between the two groups was observed regarding gender, smoking habits, thyroid function, TRAb levels, any eye features and appearance subscale score of GO-QoL. All patients had a moderate-to-severe and active GO, as defined by the 2021 EUGOGO guidelines [4], thereby requiring treatment.

**Table 1:** Demographic and clinical data at baseline

Feature	Sirolimus (*n* = 20)	Methylprednisolone (*n* = 20)	Statistics
Gender	Males 4 (20)	Males 6 (30)	OR: 0.58
	Females 16 (80)	Females 14 (70)	95% CI from 0.14 to 2.15
			*P* = 0.72*
Age (yr)	62.6 (9.8)	50.4 (13.9)	Mean difference: 12.2
			95% CI from 4.4 to 19.9
			*P* = 0.0028**
Smoking habits (no.)	Nonsmokers 12 (60)	Nonsmokers 6 (30)	Pearson’s Chi^2^: 3.67
	Ex smokers 6 (30)	Ex smokers 11 (55)	*P* = 0.15***
	Current smokers 2 (10)	Current smokers 3 (15)	
Previous treatment for GD	Methimazole 4 (20)	Methimazole 18 (90)	Pearson’s Chi^2^: 20.3
	Radioiodine 9 (45)	Radioiodine 1 (5)	*P* = 0.00015***
	Thyroidectomy 3 (15)	Thyroidectomy 1 (5)	
	Total thyroid ablation (thyroidectomy followed by radioiodine) 4 (20)	Total thyroid ablation 0 (0)	
Previous treatment for GO	None 0 (0)	None 20 (100)	OR: 0.0006
	Methylprednisolone 20 (100)	Methylprednisolone 0 (0)	95% CI from 0 to 0.031
			*P* < 0.0001*
TSH (mU/L; NV 0.4–4)	1.5 (0.7–2.5)	0.1 (0–2.4)	Mann Whitney U: 135
			*P* = 0.088†
FT4 (ng/dL; NV 0.70–1.70)	1.3 (0.3)	1.4 (0.7)	Mean difference: – 0.065
			95% CI from – 0.44 to 0.31
			*P* = 0.73**
TRAb (IU/L; NV < 1.5)	7.4 (2.2–51.025)	6.9 (3.8–9.4)	Mann Whitney U: 173
			*P* = 0.47†
GO duration (mo.)	41 (17.2–58)	8 (5.7–12)	Mann Whitney U: 40.5
			*P* = 0.00011†
Exophthalmometry (most affected eye) (mm)	23.7 (3.032)	23.1 (3.8)	Mean difference: 0.65
			95% CI from – 1.56 to 2.86
			*P* = 0.55**
Clinical Activity Score (points)	3.5 (0.7)	3.9 (0.8)	Mean difference: – 0.4
			95% CI from – 0.9 to 0.1
			*P* = 0.11**
Eyelid aperture (most affected eye) (mm)	13.1 (2.4)	13.1 (2.9)	Mean difference: – 0.050
			95% CI from – 1.78 to 1.68
			*P* = 0.95**
Sum of eye ductions (degrees)	373.4 (318.7–387.5)	351.5 (300–380.4)	Mann Whitney U: 186
			*P* = 0.61†
Diplopia (Gorman’s score)	Absent: 3 (15)	Absent: 4 (20)	Pearson’s Chi^2^: 0.45
	Intermittent: 4 (20)	Intermittent: 3 (15)	*P* = 0.92***
	Inconstant: 9 (45)	Inconstant: 8 (40)	
	Constant: 4 (20)	Constant: 5 (25)	
Best corrected visual acuity (most affected eye) (decimals)	9.4 (1.2)	9.9 (0.2)	Mean difference: – 0.5
			95% CI from – 1.086 to 0.086
			*P* = 0.092**
Quality of life total score (%)	69.032 (13.4)	79.1 (13.041)	Mean difference: – 10.072
			95% CI from – 18.56 to – 1.58
			*P* = 0.02**
Quality of life functioning subscale (%)	67.1 (18.2)	78.4 (17.2)	Mean difference: – 11.27
			95% CI from – 22.63 to 0.086
			*P* = 0.051**
Quality of life appearance subscale (%)	70.8 (16.3)	79.7 (14.8)	Mean difference: – 8.93
			95% CI from – 18.93 to 1.062
			*P* = 0.078**

Data are mean (SD), median (IQR) or *n* (%)

Statistical tests: *Fisher exact test, **ANOVA with Bonferroni’s correction, ***Pearson’s Chi square, ^†^Mann Whitney

*GD* Graves’ disease, *GO* Graves’ orbitopathy, *TSH* thyroid stimulating hormone, *FT3* triiodothyronine, *FT4* free thyroxine, *TRAbs* thyroid stimulating hormone receptor autoantibodies, *N.A.* not applicable

Median serum concentration of rapamycin in sirolimus group, measured at 12 weeks, 2–3 days before the end of treatment, was 3.9 ng/ml (IQR: 2.6–4.5), suggesting a good compliance.

**Fig. 1:**
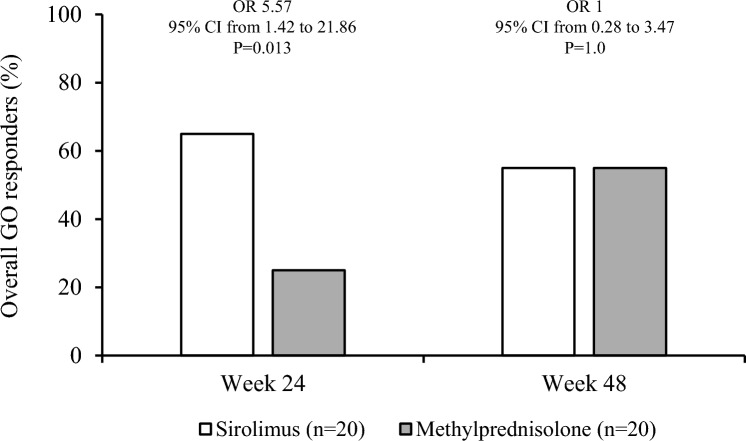
Overall outcome of Graves’ orbitopathy at 24 and 48 weeks

In confirmation of a previous study [27], the proportion of overall GO responders at 24 weeks was significantly greater in patients given sirolimus than in those treated with methylprednisolone (Fig. 1). On the contrary, the prevalence of overall GO responders did not differ between the two groups at 48 weeks (primary outcome) (Fig. 1).

**Fig. 2:**
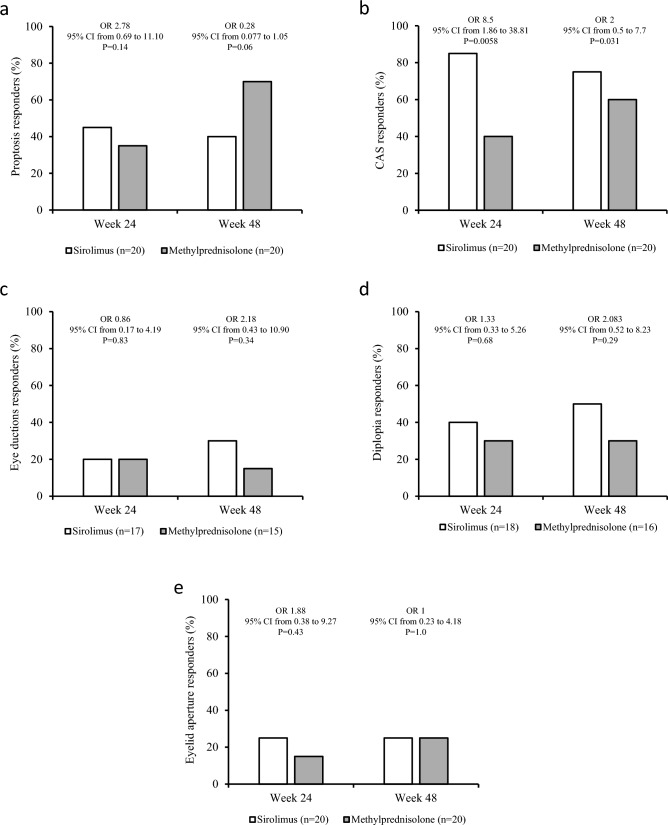
Prevalence of responders in proptosis (**a**), Clinical Activity Score (CAS) (**b**), eye ductions (**c**), diplopia (**d**) and eyelid aperture (**e**) in the sirolimus and methylprednisolone groups at 24 and 48 weeks. Prevalences were calculated on the total of patients with improvable outcome measures

**Fig. 3:**
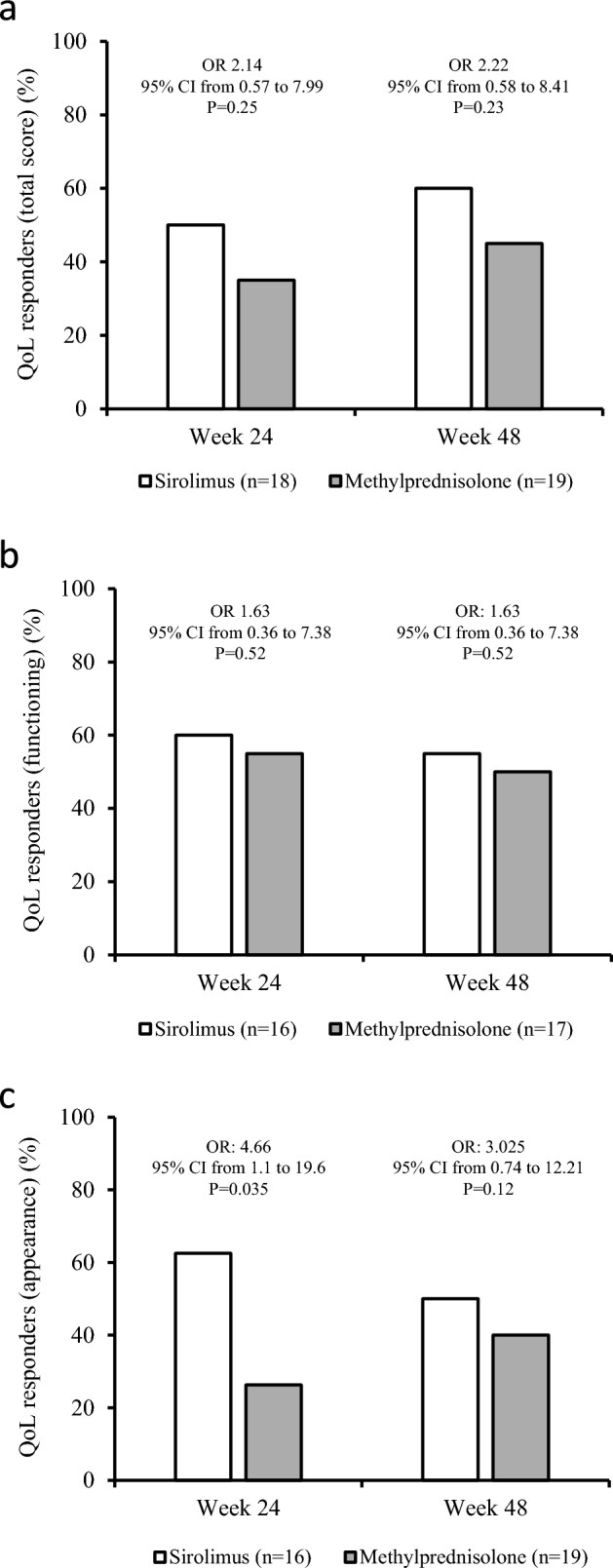
Prevalence of responders in GO-QoL total score (**a**), functioning subscale (**b**), and appearance subscale (**c**) in the sirolimus and methylprednisolone groups at 24 and 48 weeks. Prevalences were calculated on the total of patients with an improvable score

**Fig. 4:**
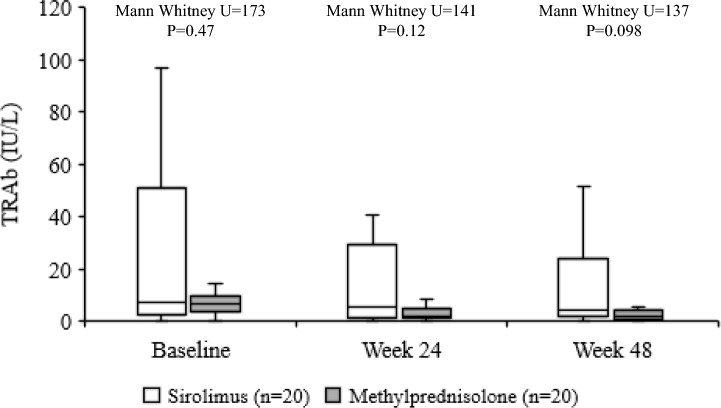
TSH-receptor antibodies (TRAb) levels in sirolimus and methylprednisolone groups at baseline, 24 and 48 weeks. Thick bars into columns represent the median values; column margins represent the intervals between the 25th and 75th percentile; error bars represent the upper and lower values

Concerning the secondary endpoints, whereas proptosis did not differ between the two groups both at 24 and 48 weeks (Fig. 2a), a higher prevalence of reduction in CAS by at least 1 point on a 5-point scale was observed in sirolimus group compared with methylprednisolone group both at 24 and 48 weeks (Fig. 2b). Eye ductions did not differ between the two groups both at 24 and 48 weeks (Fig. 2c), nor did diplopia (Fig. 2d) and eyelid aperture (Fig. 2e). Although total GO-QoL responders and responders in the functioning subscale did not differ between the two groups (Fig. 3a and b), as shown in Fig. 3c, patients treated with sirolimus had a significantly higher response rate in the appearance subscale of GO-QoL at 24 weeks, but not at 48 weeks. TRAb levels did not differ between the two groups at all time points (Fig. 4). Finally, the proportion of patients with GO relapse, defined by worsening of at least two of the eye features from 24 to 48 weeks, was not different between the two groups (not shown).

**Fig. 5:**
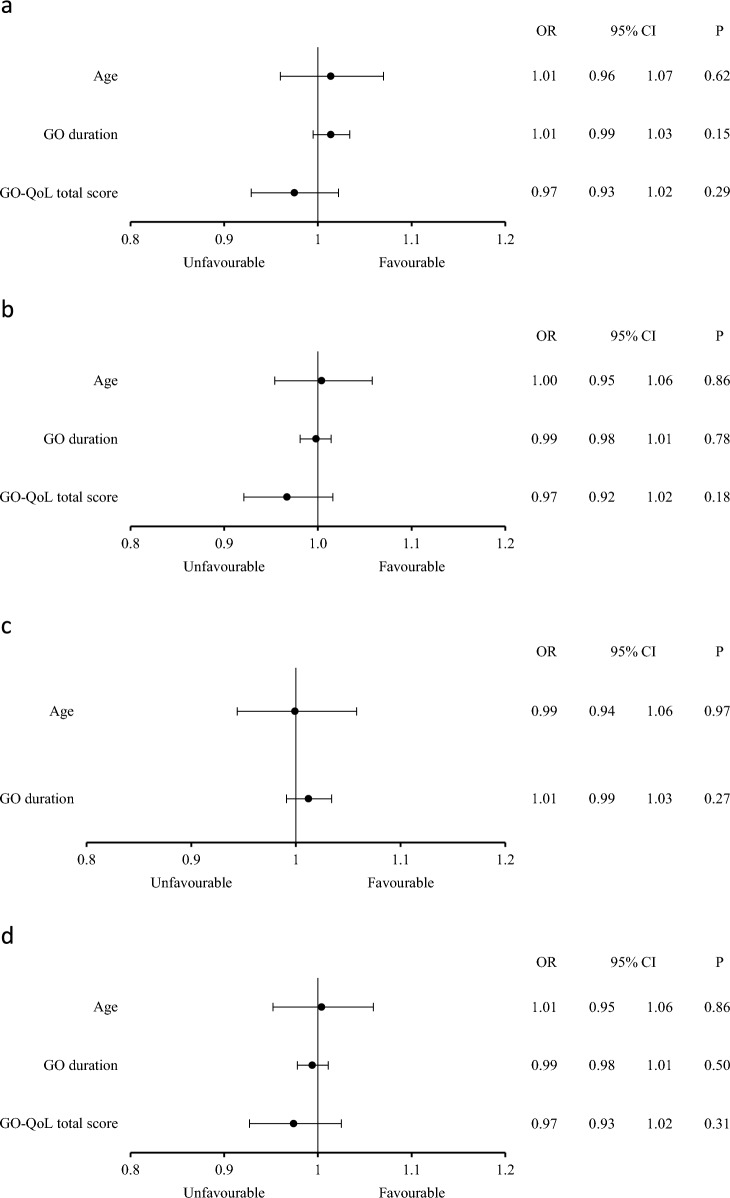
Effect of age, GO duration and GO-QoL total score on the overall outcome of GO at 24 weeks (**a**), outcome of CAS at 24 weeks (**b**), outcome of the GO-QoL appearance subscale of GO-QoL at 24 weeks (**c**) (GO-QoL total score excluded), and outcome of CAS at 48 weeks (**d**)

The features that differed between the two groups at baseline (age, GO duration and GO-QoL total score) did not affect the outcome measures (Fig. 5).

Concerning safety, at 24 weeks five mild adverse events (AEs) in 3 patients and 11 mild AEs in nine patients were recorded in sirolimus and methylprednisolone groups, respectively (Table 2). At 48 weeks, we observed six mild AEs in five patients given sirolimus and three mild AEs in three patients treated with ivGCs (Table 2). None of the reported AEs required discontinuation of the treatment or reduction in dosage.

**Table 2:** Safety

	Week 24		Week 48	
	Sirolimus (*n* = 20)	Methylprednisolone (*n* = 20)	Sirolimus (*n* = 20)	Methylprednisolone (*n* = 20)
Total	3 (15)	9 (45)	5 (25)	3 (15)
Hyperkalemia	1 (5)	0 (0)	0 (0)	0 (0)
Impaired fasting glucose	1 (5)	1 (5)	2 (10)	0 (0)
COVID-19	2 (10)	1 (5)	0 (0)	3 (15)
Flu	1 (5)	0 (0)	1 (5)	0 (0)
Arthralgias	0 (0)	0 (0)	1 (5)	0 (0)
Tachycardia	0 (0)	0 (0)	1 (5)	0 (0)
Asthenia	0 (0)	0 (0)	1 (5)	0 (0)
Hypercholesterolemia	0 (0)	1 (5)	0 (0)	0 (0)
Glaucoma	0 (0)	1 (5)	0 (0)	0 (0)
Osteoporosis	0 (0)	2 (10)	0 (0)	0 (0)
Gastroesophageal reflux disease	0 (0)	1 (5)	0 (0)	0 (0)
Epigastric pain	0 (0)	1 (5)	0 (0)	0 (0)
Weith gain	0 (0)	2 (10)	0 (0)	0 (0)
Insomnia	0 (0)	1 (5)	0 (0)	0 (0)

Data are no. of patients (%). The reported adverse events are those possibly related to treatment

## Discussion

Medical treatment of GO is challenging. EUGOGO recommends methylprednisolone as first-line treatment [4]. However, the response rate to ivGCs is variable, and several patients do not respond to treatment [5, 6]. In addition, although the intravenous route is associated with a lower risk of adverse events than oral administration, high dose ivGCs may cause several AEs, especially in patients with risk factors, namely liver or gastrointestinal diseases, hypertension, diabetes and osteoporosis [5, 6, 31, 32]. Thus, studies on alternative medications have been carried out, leading to the identification of a number of second-line treatments, namely steroid-sparing agents (cyclosporine or azathioprine) associated with oral prednisone [4], rituximab [33–35], tocilizumab [36, 37] and teprotumumab [4].

Following the reports of two GO patients resistant to glucocorticoids successfully treated with sirolimus [25, 26], we started giving sirolimus off-label in patients with moderate-to-severe and active GO in whom a previous course of methylprednisolone had failed. In a first retrospective investigation [27], as mentioned above, at 24 weeks we found a higher proportion of overall GO (composite evaluation) responders, as well as of CAS and proptosis responders, along with a better GO-QoL score, in patients treated with sirolimus compared with methylprednisolone, also given as a second line treatment.

Here we conducted a second retrospective study to assess the efficacy and safety of sirolimus in moderate-to-severe, active GO over a longer period of time, namely 48 weeks. Data from 20 patients treated with sirolimus were compared with those from 20 patients given methylprednisolone. Unlike patients given sirolimus, who had been previously treated with a course of ivGCs, patients in the methylprednisolone group were untreated, which explains why they were younger and with a shorter GO duration. Furthermore, as expected from patients with persistence or recurrence of GO after a first course of methylprednisolone, sirolimus group had a lower GO-QoL appearance score. However, none of these differences affected the results, which can be summarized as follows.

In confirmation of our previous study [27], the prevalence of overall GO responders was greater in the sirolimus group compared with the methylprednisolone group at 24 weeks (65% vs 25%). However, at 48 weeks, the overall response rate did not differ between the two groups (55% vs 55%). There was a greater prevalence of CAS responders in patients treated with sirolimus both at 24 (85% vs 40%) and 48 weeks (75% vs 60%). In addition, treatment with sirolimus was also associated with a higher response rate in appearance subscale of GO-QoL (62.5% vs 26.3%) at 24, but not at 48 weeks. GO relapse rate from 24 to 48 weeks did not differ between the two groups. Concerning the latter, it must be considered that GO duration at baseline was different in the two groups, which may have affected this parameter, as a late relapse is relatively rare in GO. All the recorded AEs were mild, and none required discontinuation of the medications or dose reduction, with no differences between the two groups.

As mentioned above, our findings substantially confirm the previously reported short-term outcome of sirolimus treatment [27]. However, in contrast with our previous findings, the prevalence of proptosis and diplopia responders at 24 weeks did not differ between the two groups, which can be explained, at least in part, by the fact that ivGCs patients included in the previous study had a longer GO duration compared to those included in the current evaluation [27 (18–48) mo. vs 8 (5.7–12) mo.]. These results were associated with a median serum concentration of rapamycin of 3.9 ng/ml, which, although slightly higher than that reported in the previous study (2.3 ng/mL) [27], is still about half of that desired in patients given sirolimus after kidney transplantation or for lymphangioleiomyomatosis [13, 14], thereby confirming that low dosage of sirolimus is sufficient to observe a beneficial effect on GO at 24 weeks. The efficacy of sirolimus can be explained by its mechanism of action, as previously reported [27].

To our knowledge, this is the first study exploring long-term outcome of sirolimus in moderate-to-severe, active GO. At 48 weeks, only CAS response rate remained significantly higher in the sirolimus group, whereas differences in overall response and in GO-QoL were attenuated. This seems to be the result of two opposite trends of response to the treatments. On one hand, it may be the consequence of a late response to methylprednisolone, which may be explained by the genomic effects of GCs, which are expected to produce clinically relevant changes within a longer period compared with non-genomic effects [32]. In this regard, it cannot be completely excluded that the previous GC treatment patients underwent to affected the response also to sirolimus, both at 24 and 48 weeks, as all patients had been previously treated with ivGCs, even though not earlier than 24 weeks before entering the study. On the other hand, responders to treatment with sirolimus in the same outcome measures decreased from 24 to 48 weeks, even though minimally, consistently with the lack of difference between the two groups concerning the GO relapse rate. Thus, we are planning to prolong treatment with sirolimus in order to assess whether a further improvement of GO could be observed at the end of a long-lasting course. If this hypothesis will be confirmed, sirolimus, as compared to methylprednisolone, could be associated with a critically better GO outcome also after 48 weeks from starting the medication.

The major limitations of the current study are its retrospective nature and the lack of randomization. However, because patients were not selected, but included by means of consecutive sampling, these limitations were partly overcome. In addition, the two study groups were different in a few features at baseline, which, however, did not affect any outcome measures, thereby guaranteeing a correct comparison between the two treatments. Furthermore, the fact that findings at 24 weeks were quite similar to those observed in our previous study [27], reassures on the reliability of the present results. Another limitation is related to the comparison of a second-line treatment (sirolimus) with a first-line therapy (methylprednisolone). However, this represented a disadvantage for the sirolimus group, thereby suggesting a more remarkably beneficial effects of sirolimus if used as first-line treatment.

In conclusion, treatment with sirolimus for three months is confirmed to be more effective than methylprednisolone in patients with GO at 24 weeks, but not at 48 weeks. Further studies are needed to assess whether the use of sirolimus as first-line treatment and/or for a longer period of time is associated with better long-term outcomes of GO.
